# Conservative Management of Unset Mineral Trioxide Aggregate Root-End Filling: A Case Report

**DOI:** 10.7508/iej.2016.03.019

**Published:** 2016-05-01

**Authors:** Masoud Parirokh, Sedigheh Farzaneh, Ali Reza Hallajmofrad

**Affiliations:** a * Endodontology Research Center, Kerman University of Medical Sciences, Kerman, Iran;*; b *Oral and Dental Diseases Research Center, Kerman University of Medical Sciences, Kerman, Iran; *; c *Private Practice, Kerman, Iran*

**Keywords:** Calcium-Enriched Mixture, Calcium Silicate, CEM Cement, Dens Invaginatus, Mineral Trioxide Aggregate

## Abstract

This case report presents conservative management of unset mineral trioxide aggregate (MTA) after being placed as a root-end filling material following periapical surgery. Periapical surgery was indicated for a maxillary lateral incisor of a 15-year-old male due to persistent exudate and a large periapical lesion. During surgery Angelus MTA was placed as root-end filling. The next session it was noticed that MTA had failed to completely set. In an orthograde approach, calcium-enriched mixture (CEM) cement was used to obturate the root canal space. The patient was followed up for 27 months and did not exhibit any clinical signs and symptoms. Radiographic images showed complete healing of the lesion.

## Introduction

Dens invaginatus is a developmental anomaly that is mostly observed in maxillary teeth, particularly in lateral incisors. The prevalence of permanent teeth with dens invaginatus has been reported to be approximately 0.3-10% [1]. It has been reported that 11.3% of the teeth with dens invaginatus exhibited pulpal pathosis [2]. The most widely used classification for dens invaginatus is introduced by Oehlers which is based on the extension of the invagination into the tooth structure [1, 3, 4].

Dens invaginatus may results in pulp necrosis and development of periradicular lesion before root-end maturation [1]. Apexification, revitalization of the pulp and placing an apical plug are among the treatment options for these teeth [5-8]. To date, no high-level evidence-based investigation has shown absolute advantage of one of these treatment options to others [8-10]. Apical plug placement may reduce the number of treatment visits and therefore decrease the treatment cost. Although apexification with calcium hydroxide has been used for treating teeth with necrotic pulps and open apices for decades, nowadays, longer treatment periods, higher costs and higher chances of the tooth fracture during the treatment make it unpopular among dental practitioners [5, 11, 12].

One of the most important aspects of endodontic practice is to provide a three-dimensional seal in the root canal space in order to prevent bacterial and their byproducts from penetrating into periradicular tissues. Gutta-percha is the most popular material used for root canal filling; however, in some instances, use of other root filling materials may be more beneficial [13].

Mineral trioxide aggregate (MTA) is a calcium silicate-based material that has excellent sealing ability and biocompatibility [14, 15]. MTA has several clinical applications, including vital pulp therapy, perforation repair, apical plug in teeth with necrotic pulps and open apices, and root canal filling [5]. The material can encourage apatite formation over its surface when kept in a synthetic tissue fluid that would increase its sealing ability [16].

MTA has several known drawbacks such as long setting time, discoloration potential, high cost and handling difficulty [5, 17]. Some attempts have been performed to overcome MTA drawbacks by adding some ingredients to the material composition [14, 18]. Meanwhile, several calcium silicate-based cements have been introduced to market such as Biodentine, calcium-enriched mixture (CEM cement), Endosequence, Endocem MTA and Bioaggregate [19, 20].

This case report presents unsuccessful MTA root-end filling in an immature maxillary lateral incisor with pulp necrosis and a large periapical lesion that was managed with an orthograde approach.

## Case Report

**Figure 1:**
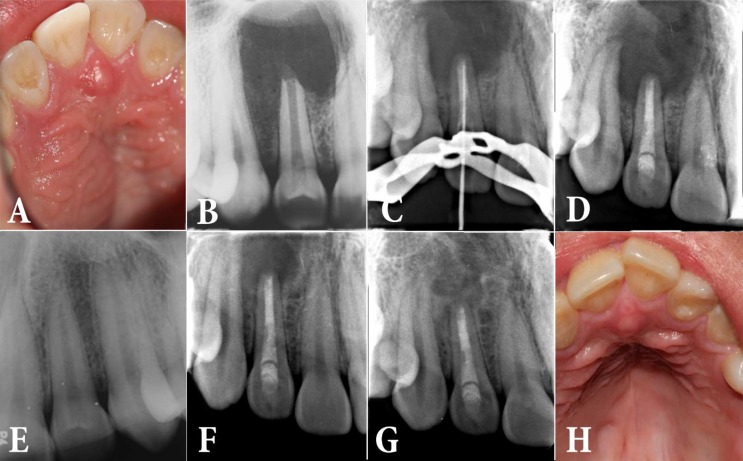
*A)* Preoperative periapical radiography of the right maxillary lateral incisor; *B)* Palatal swelling; *C)* Periapical radiography following periapical surgery; *D)* Penetration of an endodontic instrument into the root-end filling material; *E)* The radiography of left counterpart; *F)* Filling the root canal space and the unset part of Angelus MTA with CEM cement; *G)* Healed periapical lesion 27 months following the treatment; *H)* Palatal swelling has healed

A 15-year-old male attended a private dental clinic with his chief complaint being swelling in the anterior palate. His medical history was noncontributory. He mentioned no history of trauma nor a dental treatment in this region. The swelling was present for the past 2 months and the patient mentioned receiving 500 mg amoxicillin and 250 mg metronidazole 4 times per day for the last 2 weeks which were not effective on the swelling. The patient had no pain except for a mild tenderness to percussion and palpation and the maxillary right lateral incisor had no discoloration and the depth of gingival pocket was normal around all the anterior maxillary teeth (Figure 1A). The tooth exhibited no response to sensibility tests (cold, heat and electric pulp tester), while other anterior teeth had normal responses. A periapical radiography revealed a large periapical lesion and dens invaginatus in the maxillary right lateral incisor while the root apex was immature (Figure 1B). A diagnosis of pulp necrosis with acute apical abscess was made.

The treatment options, including revitalization or placement of an apical plug with a calcium silicate-based cement, were described to the patient. Because of discoloration potential of the revitalization procedure, the patient decided to choose the second treatment option. After administrating local anesthesia with 2% lidocaine containing 1:80000 epinephrine (Darupakhsh, Tehran, Iran), the tooth was isolated with rubber dam and an access cavity was prepared. Following access opening, pus drainage started from the canal. The root canal was irrigated with 5.25% sodium hypochlorite. However, the drainage it did not stop.

Attempts were made to place calcium hydroxide in the root canal but it was not successful due to continuous drainage from the root canal. The patient was instructed to change the antibiotic from amoxicillin to 300 mg clindamycin 4 times daily and the next session was scheduled one week later. The procedure was repeated with no success because of persistent active exudate which interfered with placement of calcium hydroxide in the canal. Therefore, two treatment options including either decompression by placement a drain in the palate to gradually reduce the size of the lesion or surgical removal of the lesion followed by the placement of the root-end filling during surgery, were presented to the patient and his parents. They opted for surgery.

According to the surgeon, the surgery included a flap reflection and placement of white Angelus MTA (MTA-Angelus, Angelus, Londrina, PR, Brazil) for root-end filling after apical root resection. The surgeon again referred the patient to our clinic for obturation of the remaining unfilled root canal space over the root-end filling. In the treatment visit, a periapical radiography showed the presence of a radiopaque root-end filing material. After administration of 0.9 mL of lidocaine with 1:80000 epinephrine and placing a rubber dam, the access cavity was reopened and setting of the root-end filling was checked. Unfortunately, the root-end filling material did not completely set and the file could penetrate into the material. The largest root canal instrument that contacted the root canal wall and the root-end filling material was placed in the canal. A #60 file was suitable for inserting into the canal. Root ZX apex locator (J. Morita Corporation, Kyoto, Japan) was used to make sure that the file did not penetrate beyond the root canal space. As soon as the apex locator alarm was heard, file penetration was stopped and a periapical radiography was taken. The radiographic image showed that the file penetrated through the root-end filling material (Figure 1C). Because of incomplete root-end closure and low thickness of the root it was decided to place a fresh calcium silicate material inside the root canal space instead of trying to remove the remaining root-end filling material. Therefore, the space of unset root-end filling material was prepared to #80. Thereafter, root canal space was dried with sterile paper points and CEM cement (BioniqueDent, Tehran, Iran) was used to fill the entire length of the root canal. A wet cotton pellet was placed over the cement. The access cavity was sealed with a temporary sealing material (Provis, Favodent, Karl Huber GmbH, Germany).

One week later, the tooth was reopened and setting of CEM cement was evaluated. After making sure of the cement setting, the patient was referred for final restoration of the access cavity (Figure 1D). Because of possible bilateral involvement, a periapical radiography was taken and pulp sensitivity tests were performed for the left maxillary lateral incisor, as well (Figure 1E). The test results were normal and the periapical radiography did not reveal any periapical pathosis. After three months the mild palatal firm swelling was still present; however, the size of the periapical lesion had decreased considerably (Figure 1F). Recalling the patient up to 27 months following the treatment showed complete clinical recovery, and the lesion exhibited healing in periapical radiography (Figure 1G and 1H).

## Discussion

This case report presented a case of incomplete setting of white Angelus MTA following root-end filling that was successfully treated with CEM cement without removing all the root-end filling material during orthograde retreatment. It has been shown that dens invaginatus might be symmetrical [21]. Thus, in the present case the left maxillary lateral incisor was also checked for the presence of dens invaginatus and pulp pathosis by performing sensibility tests and taking a periapical radiography.

The first option for treating a large periapical lesion is to perform orthograde root canal treatment [22]. In the present case, it was not possible to perform orthograde treatment because of persistent and active exudation. In this condition, the practitioner might either perform periapical surgery or decompression [23, 24].

One of the major drawbacks of MTA was the material’s long setting time [5]. Laboratory studies have shown that acidic pH and adding some ingredients to calcium silicate-based materials may compromise their setting [25, 26]. Blood contamination has also had some impacts on the physical properties of calcium silicate-based cements [27], particularly when the material is placed as root-end filling with low thickness. In addition, dry conditions may adversely affect the material’s setting [28]. Fetal bovine serum also prevented MTA setting in a laboratory study [29]. Several investigations have shown the failed setting of calcium silicate-based cements in certain conditions such as addition of chlorhexidine to MTA [25] and placing CEM cement in an acidic environment [26].

The manufacturer of Angelus MTA has claimed that the material has shorter setting time compared to the original form of ProRoot MTA due to the absence of dehydrated calcium sulfate [14]. There are several reasons that MTA may not set after mixing, including the presence of acidic pH, specific types of surrounding environment, blood contamination when low thickness of the material is placed in the root canal, and incorporation of some ingredients into the material’s composition [25, 29-31]. A recent investigation confirmed that keeping MTA in an acidic environment may have some influence on crystalline structures of the material [32].

In addition, as the treatment had been performed by two different practitioners there is a little chance that technical problems during the material placement or contamination might have led to unsetting of Angelus MTA. It has been confirmed that calcium silicate-based materials are very technique sensitive cements[14].

A study reported that two most important reasons for surgical failure are incorrect root-end preparation and no placement of root-end filling [33].

Low thickness of MTA as root-end filling material may be another reason for the material’s failure to set. MTA setting may be compromised even in 2-mm thicknesses where the bulk is contaminated with blood [30]. However, in this case, the periapical radiography showed that the material thickness was more than 2 mm.

It has been shown that blood contamination during MTA placement might compromise the material’s setting [34-36]. Based on pathologic report the lesion was a radicular cyst; however, blood contamination was likely during the surgical procedure. It has been recommended that when periapical surgery is going to be performed for a tooth with a large periapical lesion, root-end resection and placement of root-end filling material should be performed prior to the lesion curettage in order to prevent blood contamination of the root-end filling material [37].

In the present case, CEM cement was used as a root canal filling material instead of MTA. CEM cement is calcium silicate-based cement with excellent sealing ability and high biocompatibility with the same clinical applications of MTA [38-40].

In this case, the authors decided to keep the set part of the root-end filling material and did not try to completely remove it. There are several reasons why the authors made that decision. Firstly, complete removal of MTA from the root canal was not possible. Secondly, the root canal wall thickness was very low and therefore trying to remove previously placed root-end filling material could adversely affect the tooth structure, making it susceptible to further fracture. Thirdly, there was a possibility that previously placed root-end filling material or the fresh material that was going to be placed as the apical plug might have been pushed into the periradicular area. It has been shown that extrusion of dental materials and even MTA might have adverse effects on periapical healing [41].

In this case, use of two different calcium silicate-based cements for treating an open apex tooth resulted in healing. This protocol may help practitioners in decision making where no high level of evidence is present. Future laboratory studies and *in vivo* investigations can be performed to collect more information regarding the behavior of calcium silicate cements when they have to be placed in conditions like the present case.

## Conclusion

This case report presented successful conservative orthograde treatment of an open apex tooth when white Angelus MTA failed to set completely after being used as a root-end filling. Using CEM cement for repairing the unset part of white Angelus MTA and 27-month follow up confirmed treatment success.
